# Development and Assessment of Heavy Oil-Degrading Fungal Consortia (*Aspergillus* and *Alternaria*) for Soil Bioremediation

**DOI:** 10.3390/jof12030224

**Published:** 2026-03-19

**Authors:** Shujuan Peng, Junhao Zhu, Weiguo Liu, Junhui Zhang

**Affiliations:** Key Laboratory of Oasis Ecology of the Ministry of Education, College of Ecology and Environment, Xinjiang University, No. 777 Huarui Street, Urumqi 830046, China

**Keywords:** heavy oil biodegradation, synthetic consortia, fungal genomics, metabolic mechanisms, bioremediation

## Abstract

Leveraging fungal consortia to degrade heavy oil is an emerging strategy for mitigating/cleaning up environmental pollution. However, many consortia are predominantly evaluated by measuring the biodegradation efficiency of heavy oil, with insufficient attention paid to the mechanistic underpinnings and metabolic pathways. In this study, heavy oil-degrading fungal consortia were developed for potential application in soil bioremediation. Whole-genome sequencing was used to predict the metabolic pathways and interspecific interactions driving heavy oil biodegradation. Three heavy oil-degrading fungal strains, designated *Aspergillus corrugatus* FH2, *Aspergillus terreus* FL4, and *Alternaria alstroemeriae* FW1, were isolated from oil sludge in the Karamay Oilfield in Xinjiang, China. Four consortia were constructed through the combination of two or three strains. The consortium F13 (FH2 + FW1) achieved 72.0% removal of heavy oil in a simulated bioremediation test over 30 days, which was more efficient than other consortia and single strains (59.5–68.5%). Notably, the mean degradation rate of long-chain alkanes (C_24_–C_28_) by F13 reached 95.9%. After F13 treatment, the major fractions of heavy oil showed considerable degradation, 87.4% for saturates, 92.0% for aromatics, 69.5% for resins, and 27.3% for asphaltenes. Genome annotation of FH2, FL4, and FW1 revealed the presence of core genes for degradation of *n*-alkanes and aromatics, e.g., *CYP505*, *frmA*, *fadB*, *hmgA*, *ALDH*, and *ACSL.* These functional genes encoded cross-lineage enzymes, enabling synergistic catabolism of C_13_–C_28_ alkanes and aromatics. Our findings indicated that the fungal consortium of *A. corrugatus* FH2 and *Al. alstroemeriae* FW1 has remarkable bioremediation potential for heavy oil-contaminated sites. This study provides molecular evidence for the design of targeted interventions to improve soil remediation efficiency with fungal consortia.

## 1. Introduction

Heavy crude oils rich in asphaltenes have drawn global attention due to the rapid depletion of conventional light oils and the accelerating growth of energy demands [1]. To alleviate the energy pressure, the petroleum industry will continuously increase heavy oil production in the coming years [2]. Unfortunately, a huge amount of heavy oil is leaked and spilled into the environment during the exploration and production, as well as transportation, refining, and distribution processes. The introduction of petroleum hydrocarbons into soil deteriorates soil properties, leading to delayed seed germination and reduced plant emergence. Soil contamination with petroleum hydrocarbons additionally affects the survival of arthropods (e.g., earthworm) and impairs microbial growth [3]. Due to their higher hydrocarbon structural complexity, larger viscosity, and greater resistance to degradation, heavy oils can cause more serious and lasting damage to the soil environment than conventional crude oils [4]. This underscores the need for innovative and efficient remediation strategies tailored to address heavy oil contamination in green and safe manners.

Bioremediation is recognized as one of the most preferred strategies for cleaning up oil from contaminated sites given its low cost and high ability to convert organic pollutants to harmless end-products [5]. This environment-friendly technique employs specialized microorganisms that are able to biodegrade organic pollutants from contaminated sites into simpler, less toxic molecules (e.g., CO_2_, H_2_O) under suitable conditions. Importantly, bioremediation enables complete degradation of pollutants, while other conventional methods leave toxic secondary by-products [6]. In the past few decades, multifarious microorganisms (including bacteria, fungi, and microalgae) capable of degrading heavy oil and adapting to harsh environments have been isolated from oil-contaminated locations [7]. Fungi, thanks to their robust and unique biodegradation actions, seem to be particularly promising for metabolizing heavy and extra-heavy oils [8]. Among the fungi isolated from contaminated areas, *Aspergillus*, *Penicillium*, and *Trichoderma* secrete important oxidative enzymes, such as laccases (LaC), manganese peroxidases (MnP), lignin peroxidases (LiP), and versatile peroxidases (VP). These enzymes catalyze the oxidation of numerous saturates and aromatics, facilitating the degradation of different hydrocarbons [9]. Non-specific fungal enzymes (e.g., LaC, MnP, LiP, VP) are more effective than bacterial enzymes (e.g., alkane hydroxylases, cytochrome P450 [CYP] oxygenases, dioxygenases) in breaking down recalcitrant hydrophobic compounds [10,11]. Additionally, the hyphal network of filamentous fungi exhibits apical growth, allowing them to penetrate the soil matrix more efficiently than bacteria. This mechanical force drives fungi to reach inaccessible interstices where pollutants are difficult to remove [9]. An example is provided by Silva Monteiro et al. [12], who reported that the fungal strain *Penicillium janthinellum* P05R3 achieved higher degradation rates (82.5%, 83.2%) of total petroleum hydrocarbons (TPHs, C_5_–C_40_) and polycyclic aromatic hydrocarbons (PAHs) compare to the bacterial strain *Stenotrophomonas maltophilia* P05R11 (46.0%, 36.6%).

Despite the advantages of fungi over bacteria for biodegradation of high-molecular-weight (HMW) hydrocarbons, two principal factors limit their application in bioremediation of contaminated soils [13]. First, heavy oil consists of various complex hydrocarbons, and a single strain can degrade only a limited range of hydrocarbons. Secondly, low hydrocarbon bioavailability and non-optimal environmental conditions affect the biodegradation efficiency of microorganisms [14]. To tackle these issues, researchers developed consortia of filamentous fungi and/or bacteria capable of degrading petroleum hydrocarbons. They emphasized the superiority of microbial consortia over single strains in pollutant removal and soil colonization through co-metabolism or metabolic cooperation, including cross-feeding and metabolic exchange (e.g., “hitchhiking” strategy) [9]. In addition to exhibiting greater competitiveness than individual strains, microbial consortia also secrete extracellular substances, such as polysaccharides, proteins, and enzymes. These substances can act as nutritional resources to foster metabolic cooperation and improve microbial adaptability, contributing to efficient degradation of oil pollutants [15]. Thus, microbial consortia offer an efficient solution to degradation and removal of heavy oil pollutants.

Two major strategies can be used to construct microbial consortia for bioremediation. One method is bottom-up co-culture, which is the artificial combination of a limited number of pure strains with distinct functions. The other method is top-down selection and enrichment, whereby the consortia in environmental samples are enriched and selected by subculturing [16]. Compared to top-down consortia, bottom-up consortia are simpler in composition and clearer in functional division. Additionally, the structure of bottom-up consortia can be modified and optimized for different target products [16,17]. When constructing microbial consortia to degrade heavy oil pollutants, an important step is to select suitable chassis strains with desired catalytic performance. Whether the selected strains can coexist with other strains also needs to be considered. Both the genera *Aspergillus* and *Alternaria* are soil-inhabiting saprotrophic fungi, which have been frequently reported as potent degraders of crude oil and its major fractions, including aliphatic hydrocarbons and PAHs [8]. Ramoutar et al. [18] observed 76.0% degradation of crude oil by a fungal consortium that contained *Aspergillus terreus*, *Fusarium proliferatum*, *Penicillium* sp., and *Aspergillus* sp. isolated from oil impacted soil in La Brea Pitch Lake. Recently, Romero-Hernández et al. [19] have isolated *Aspergillus sydowii* and *A. terreus* isolated from hydrothermal vents of the Pescadero Basin, which are able to degrade 40.6% and 16.0% of extra-heavy oil, respectively. Genomic studies reveal that both *Aspergillus* and *Alternaria* harbor *adhP*, *ALDH*, and *CYP* genes encoding catabolic enzymes, like ethanol dehydrogenases, aldehyde dehydrogenases, and CYP monooxygenases. These functional genes play a vital role in the biodegradation and removal of heavy oil pollutants in soils [9]. The previous findings indicate the feasibility of developing heavy oil-degrading consortia using *Aspergillus* and *Alternaria* strains for soil remediation.

Myriad studies have highlighted the biodegradation efficiency and application prospect of fungal consortia, which show high tolerance to hydrocarbon toxicity and outstanding ability to synthesize cassettes of non-specific enzymes [20]. However, there is still a paucity of investigations on gene interactions involved in fungal biodegradation of heavy oil. In particular, the core genes and possible metabolic pathways for petroleum hydrocarbon biodegradation are poorly understood. Identifying strain genotypes and revealing their metabolic interactions are crucial for understanding the potential suitability and stability of fungal consortia for biodegradation of heavy oil pollutants [21]. Many researchers assessed the hydrocarbon degradation potential of microorganisms indirectly relying on phenotypic and biochemical information, without prior identification of their genotypes [22]. Rapid development of whole-genome sequencing technology has unlocked possibilities to determine microbial functional genes responsible for heavy oil biodegradation. The functional capabilities of heavy oil-degrading fungi are governed by the expression of specific genes. The key genes responsible for petroleum hydrocarbon biodegradation in each strain can be identified through whole-genome sequencing and annotation. Furthermore, genomic analysis can be adopted to uncover microbial metabolic pathways and interspecific interactions driving heavy oil biodegradation [23]. It is vital to elucidate the molecular mechanisms of core gene-mediated biodegradation of heavy oil by fungal consortia. This will enable the design of targeted microbial interventions to improve the biodegradation efficiency of heavy oil pollutants and develop contingency plans for oil spill response.

The objectives of this study were to (1) isolate heavy oil-degrading fungi from oil sludge and construct consortia based on the biodegradation activity of pure strains; (2) verify the biodegradation performance of fungal consortia by analyzing the degradation of *n*-alkanes and removal of petroleum hydrocarbons in simulated soil; and (3) pinpoint the functional genes and synergistic mechanisms of fungal consortia for heavy oil biodegradation through whole-genome sequencing. Results of this study could reveal how synthetic fungal consortia achieve efficient biodegradation of heavy oil at the gene level. Our research will pave the way toward the construction of high-performance fungal consortia and their application in bioremediation of heavy oil-contaminated soils.

## 2. Materials and Methods

### 2.1. Heavy Oil, Oil Sludge, and Culture Media

The heavy oil and oil sludge used in the study were collected from Karamay Oil Field in Xinjiang, Northwest China. The heavy oil had a viscosity of 12,658 mPa·s at 50 °C, which was used as a carbon source for fungal isolation and biodegradation tests. The heavy oil sludge contained 83.0 mg g^−1^ of TPH and served as a source of fungi for enrichment and isolation. For details of heavy oil and oil sludge properties, see our previous publication [24]. A basal mineral salts medium (MSM) was prepared as previously reported [25] with minor modifications. The MSM contained 2.00 g of urea, 0.50 g of MgSO_4_·7H_2_O, 5.00 g of NaCl, 2.00 g of KH_2_PO_4_, and 3.00 g of K_2_HPO_4_·3H_2_O per liter tap water. The screening and biodegradation medium was formulated by adding 40.0 g of heavy oil and 10 g of agarose to the MSM. Fungal isolation, purification, and preservation were performed using the potato dextrose agar (PDA) medium. Briefly, 200.0 g of potato tuber was washed, peeled, and cut into small pieces. The potato pieces were boiled in 1000 mL of tap water for 20–30 min and then filtered through gauze. Ten grams of agar was added to the potato extract and completely dissolved, followed by the addition of 20.0 g of dextrose. The total volume of the PDA medium was brought up to 1000 mL with tap water. All chemical reagents used in the study were purchased from Xinbote Chemical Reagent Co., Ltd., Tianjin, China. All media were sterilized (121 °C, 30 min) and their pH was adjusted to 7.0 before use.

### 2.2. Fungal Isolation, Screening, and Identification

Heavy oil-degrading fungi were enriched and isolated from the heavy oil sludge based on the method of Romero-Hernández et al. [9] with slight modifications. Briefly, a 10.0 g sample of the oil sludge was dispersed in 100 mL of sterilized MSM supplemented with 2.0% heavy oil as sole carbon source. The sample was incubated on a rotary shaker at 120 r min^−1^ for 7 days at 30 °C. The enrichment culture was 10-fold diluted to 10^−4^ and 0.05 mL of each dilution was spread onto duplicate PDA plates. The inoculated plates were incubated at 30 °C for 7 days. Fungal colonies with distinct morphologies and colors were selected, purified, and preserved on PDA slants (4 °C). All purified isolates were spotted onto oil plates containing MSM supplemented with 40.0 g L^−1^ of heavy oil and incubated at 30 °C for 7 days. Based on their superior growth performance on heavy oil plates (visual growth and quantitative diameter measurements), three isolates (designated FH2, FL4, and FW1) were selected as potential candidates for consortium development and subsequent assays. Identification of the three isolates was conducted by observation of colony and microscopic morphologies coupled with DNA sequencing [26]. The internal transcribed spacer (ITS) rDNA sequences of the fungal isolates obtained in this study have been submitted to the NCBI GenBank with accession numbers PX397388 (FH2), PX397387 (FL4), and PX399777 (FW1).

### 2.3. Optimal Co-Substrate Selection

The addition of co-substrates, such as soluble carbon sources can stimulate fungal growth. The presence of co-substrates can also upregulate the production of crucial enzymes for degrading recalcitrant hydrocarbons [12]. In this study, sucrose, glucose, or soluble starch (0.2% each, *w*/*v*) was used as an organic carbon source for heavy oil-degrading fungi, and yeast extract (0.2% each, *w*/*v*) was used as an organic nitrogen source. The selected strains were separately inoculated onto the screening medium added with the co-substrates and incubated at 30 °C for 5 days. The growth diameter of colonies was recorded daily and three replicates were included for each group. The organic carbon source that was most effective in promoting colony growth was selected as the optimal co-substrate.

### 2.4. Fungal Consortia Development and Optimization

Three fungal strains that showed outstanding abilities to utilize heavy oil were selected to develop synthetic consortia. Ninety-six-hour broth cultures of two or three strains were mixed in equal ratios to obtain four consortia, F12 (5 mL of FH2/FL4), F13 (5 mL of FH2/FW1), F23 (5 mL of FL4/FW1), and F123 (3.5 mL of FH2/FW1 and 3.0 mL of FL4). Then, the mixed cultures (10 mL each) were transferred into 100 mL of fresh liquid PDA medium and incubated for 5 days. The cell biomass of fungal consortia and their growth diameters on heavy oil plates were, respectively, measured using the methods of Yang et al. [25] and Zhang et al. [27]. The potential interactions between fungal strains were classified based on the growth performance of the consortium relative to single strains. When a consortium demonstrated higher biomass yield and larger colony diameter than the average of single strains, the interactions between strains were identified as compatibility or synergy; otherwise, the interactions between strains were identified as antagonism.

### 2.5. Heavy Oil Removal Test

The high content of recalcitrant components and dense texture of heavy oil could limit the effect of direct bioremediation. Thus, an inorganic bulking agent (find sand) was added to the heavy oil for structural modification, and an organic bulking agent (wheat bran) was added to enhance water retention in the culture medium. Fine sand was used as a substitute for natural soil taking into account site representativeness and oil extractability. First, the heavy oil sludge samples were collected from a field site with a high proportion of sandy soil, making sand a relevant matrix for simulating the native environment of the isolates. Second, preliminary tests showed that heavy oil could be mixed more uniformly with sand than with soil. Wheat bran was incorporated into the simulated soil to increase the porosity and aeration of the sand-based system. This could enhance oxygen availability—a critical factor for aerobic fungal metabolism. Additionally, wheat bran provided a natural surface for fungal attachment and colonization, promoting biofilm formation and overall microbial activity.

The simulated soil bioremediation test was conducted in 600-mL tissue culture vessels. Each vessel contained 45 mL of MSM supplemented with 2.00 g of heavy oil, 5.00 g of wheat bran, and 50.00 g of fine sand (44.1% moisture). Sucrose (2 g·L^−1^) and yeast extract (2 g·L^−1^) were, respectively, used as auxiliary carbon and nitrogen sources. After sterilization (121 °C, 30 min) and cooling, the bioremediation medium was inoculated with 10% mixed or pure cultures and then incubated at 30 °C 30 days under aerobic conditions. The medium without inoculum served as the control, with three replicates per treatment. The degradation rate of heavy oil was determined gravimetrically. Alumina column chromatography was used to quantify saturates, aromatics, resins, and asphaltenes (SARA) contents in the heavy oil after biodegradation [28]. The composition of original and residual oil samples was analyzed by gas chromatography–mass spectrometry (Pegasus 4D^®^/7890B; LECO, St. Joseph, MI, USA) [24].

### 2.6. Whole-Genome Sequencing

The three fungal strains were activated on PDA slants, transferred into 100 mL of liquid PDA medium, and then incubated at 30 °C shaken at 120 r min^−1^ for 5 days. After that, the culture broths were centrifuged at 10,000 rpm for 10 min at 4 °C to pellet cells. The cell pellets were washed twice with phosphate-buffered saline (0.1 M, pH 6.8) and snap frozen in liquid nitrogen for 10 min. Samples were kept on dry ice for delivery to Benagen Technology Co., Ltd. (Wuhan, Hubei Province, China), where fungal genomes were sequenced by Oxford Nanopore sequencing on the PromethION platform using R9.4.1 flow cells, controlled by MinKNOW software (version 23.07.12). After sequencing, repetitive sequences were removed from the raw reads and local re-alignment was performed. NECAT v0.01 (https://github.com/xiaochuanle/NECAT, accessed on 2 February 2026) was used to assemble the final reads into a draft genome. Subsequently, structural prediction of repetitive elements, protein-coding genes, tRNA genes, and rRNA genes within the assembled genomic sequence was conducted. The repetitive elements, including tandem repeat and interspersed repeat sequences, were predicted using the Tandem Repeats Finder (version open-4.0.9) and RepeatMasker (version open-4.0.9) (http://www.girinst.org/repbase, accessed on 2 February 2026). GlimmerHMM (http://ccb.jhu.edu/software/glimmer/index.shtml, accessed on 2 February 2026) was used to predict the protein-coding genes. The tRNA and rRNA genes were predicted using tRNAscan-SE (version 1.23) and rRNAmmer (version 1.23), respectively. For functional annotation of the genes, the following four databases were used, NR (Non-Redundant Protein Database, version 2024-02-07; https://ftp.ncbi.nlm.nih.gov/blast/db/FASTA/, accessed on 2 February 2026), GO (Gene Ontology, version 2024-05-29; http://geneontology.org/, accessed on 2 February 2026), KOG (Cluster of Orthologous Groups of eukaryotic complete genomes, version 5.0; http://eggnog5.embl.de/#/app/home, accessed on 2 February 2026), and KEGG (Kyoto Encyclopedia of Genes and Genomes; version 2024-05-27) (https://www.kegg.jp/kegg/, accessed on 2 February 2026). The CAZy (Carbohydrate-Active enZymes, version dbCAN2 V12; http://www.cazy.org/, accessed on 2 February 2026) database was used to annotate the functions of enzymes involved in carbohydrate biosynthesis and metabolism.

### 2.7. Statistical Analysis

The experimental data are presented as the mean ± standard deviation of three replicates. Statistical analyses were performed using SAS (version 9.2; SAS Institute Inc., Cary, NC, USA). Multiple comparisons of group means were carried out using one-way analysis of variance (ANOVA) followed by Duncan’s multiple-range test (*p* < 0.05). Graphs were created using Origin (version 2022; OriginLab Corp., Northampton, MA, USA).

## 3. Results and Discussion

### 3.1. Screening and Identification of Heavy Oil-Degrading Fungi

#### 3.1.1. Isolation and Screening of Fungal Strains

**Figure 1:**
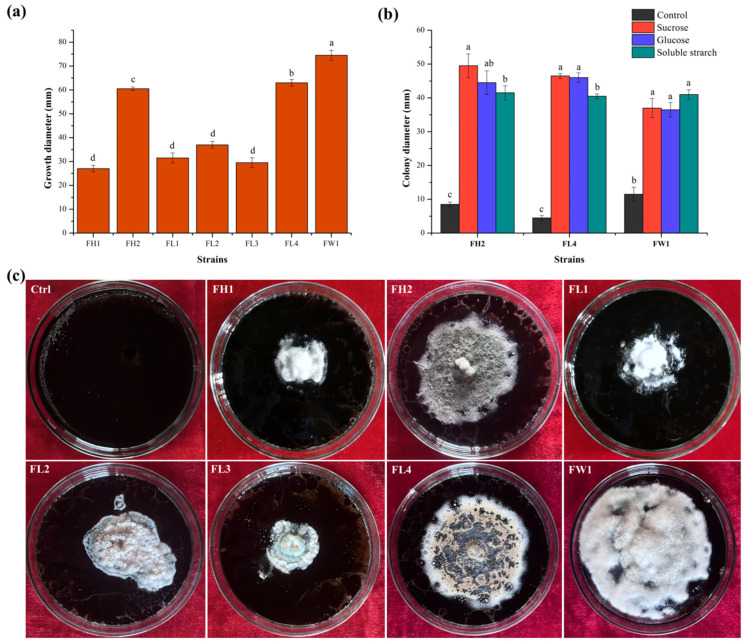
Growth diameter (**a**), colony diameter (**b**), and growth performance (**c**) of five fungal isolates on the basal mineral salt medium containing heavy oil (40.0 g·L^−1^) after 7 days of incubation. Error bars represent the standard deviation of the mean (*n* = 3). Significant differences between groups are indicated by different lowercase letters above the error bars based on Duncan’s multiple range test (*p* < 0.05).

Seven heavy oil-degrading fungi, designated FH1, FH2, FL1, FL2, FL3, FL4, and FW1, were isolated from oil sludge in the Karamay Oilfield through enrichment culture. The ability of these fungal isolates to utilize heavy oil was preliminarily demonstrated by their growth performance in oil plates. While all seven isolates were able to grow with heavy oil as the sole carbon source, FH2, FL4, and FW1 showed superior growth compared to FH1, FL1, FL2 and FL3 (Figure 1c). Among them, FW1 produced the largest growth diameter (74.5 mm), followed by FL4 (63.0 mm) and FH2 (60.5 mm), indicating their outstanding oil utilization ability (*p* < 0.05). FH1, FL1, FL2 and FL3 exhibited a lower ability to utilize heavy oil compared to other isolates, as indicated by smaller growth diameters (27.0, 31.5, 37.0 and 29.5 mm, respectively; Figure 1a). The biodegradation capability of microorganisms allows them to grow on heavy oil as a sole carbon source, producing a variety of bioproducts [14]. Based on their growth performance on heavy oil, three fungal isolates (FH2, FL4, and FW1) were screened out as possible heavy oil degraders.

Heavy oil contains a vast range of aliphatic, aromatic, and polynuclear hydrocarbon molecules. Fungi have been recognized as promising tools for remediating hydrocarbon-contaminated soils owing to their strong and peculiar biodegradation actions on hydrocarbons [29]. In principle, fungi are advantageous to bacteria for their powerful enzymatic systems, as well as rapid adaptation to toxic pollutants and harsh environmental conditions. This can be exemplified by the superior capability of *P. janthinellum* P05R3 compared to *S. maltophilia* P05R11 for removing a broad range of hydrocarbons from contaminated soils [12]. To date, a huge number of fungal strains isolated from contaminated sites have been shown to grow well on complex hydrocarbon mixture as the sole carbon source, demonstrating their adaptability to polluted environments [30]. A high proportion of the known hydrocarbon-degrading fungi belong to the phylum Ascomycota (including *Aspergillus*, *Penicillium*, and *Alternaria*), less assigned to Basidiomycota and very rarely to other phyla (e.g., *Mucoromycota*, including *Rhizopus*, *Mucor*, and *Cunninghamella*) [31]. Recently, Romero-Hernández et al. [19] have isolated *Aspergillus*, *Penicillium*, and *Alternaria* strains from deep-sea sediments in the vicinity of hydrothermal vents, demonstrating their high biodegradation efficiency for extra-heavy oil. In our study, three fungal isolates (FH2, FL4, and FW1) were obtained from the oil sludge, which could efficiently utilize heavy oil and exhibit higher growth performance than other isolates. Our findings highlight the significant potential of specific fungal isolates for heavy oil biodegradation, especially below the temperature of 30 °C.

#### 3.1.2. Morphological and Molecular Identification of Fungal Strains

**Figure 2:**
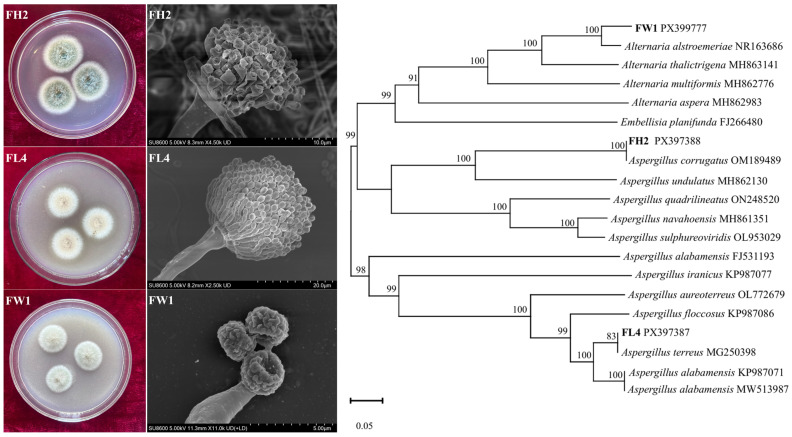
Morphological features (**left**) and phylogenetic tree (**right**) of three efficient heavy oil-degrading fungal strains (FH2, FL4, FW1).

Next, the three fungal isolates were identified on the basis of morphological observations and ITS gene sequencing. Figure 2 illustrates the distinct colony and microscopic morphologies of the three isolates. FH2 formed filamentous colonies with a ~2.8 cm diameter, a rough, raised surface, and grayish green conidia. FL4 developed filamentous colonies with a ~2.3 cm diameter, a rough, flat surface, and brown conidia. FW1 formed filamentous colonies with a ~2.0 cm diameter, a rough, raised surface, and black-brown conidia. Scanning electron microscopy images revealed that FH2 and FL4 produced globular spores with rough and smooth surface structures, respectively; FW1 formed oval spores with rough surface structure. Molecular identification of the isolated fungi was performed by sequencing the ITS rDNA region. The obtained sequences were aligned with available sequence data in the GenBank using NCBI BLAST (https://blast.ncbi.nlm.nih.gov/Blast.cgi, accessed on 2 February 2026) to construct a phylogenetic tree (Figure 2). FH2 shared the highest sequence identity (100.0%) to *Aspergillus corrugatus* PX397388. FL4 was highly related to *Aspergillus terreus* PX397387 (100.0% sequence identity). FW1 was a close relative of *Alternaria alstroemeriae* PX399777 (100.0% sequence identity). Based on their colony morphologies, microscopic characteristics, and ITS sequences, FH2 was identified as *A. corrugatus*, FL4 was identified as *A. terreus*, and FW1 was identified as *Al. alstroemeriae*.

*Aspergillus* and *Alternaria* are two fungal genera commonly found in soils, both of which have been reported as petroleum hydrocarbon degraders and proven to be able to degrade up to 57% of hydrocarbons [32]. Particularly, *Alternaria* species have been associated with the biodegradation of heavy oil and high-molecular-weight PAHs [8]. Members of *Alternaria*, an extremely diverse and ubiquitous genus, are able to secrete extracellular enzymes (including LaC and peroxidases), which promote the oxidation and decomposition of recalcitrant PAHs [26]. Furthermore, the frequent isolation of *Aspergillus* and *Alternaria* species from contaminated sites likely indicates their adaptation under petroleum hydrocarbon contamination [8,26,32]. Our fungal strains *A. corrugatus* FH2, *A. terreus* FL4, and *Al. alstroemeriae* FW1 all belong to the phylum Ascomycota and exhibit remarkable growth with heavy oil as the only carbon source (Figure 1c). Hence, these three strains can be used to construct heavy oil-degrading consortia for simulated soil bioremediation tests.

### 3.2. Optimization of Co-Substrates for Heavy Oil Biodegradation

The vast majority of fungi are unable to utilize petroleum hydrocarbons as the only source of carbon and energy. Nevertheless, many fungi can co-metabolize hydrocarbons into smaller organic compounds and, in some cases, CO_2_ [7]. Glucose, sucrose, and soluble starch are often used as co-substrates to heighten microbial activity, thereby enhancing cell growth and breakdown of recalcitrant hydrocarbons [33,34]. For instance, glucose-supplemented media have been shown to boost fungal growth and LaC production, which in turn accelerates the biodegradation of long-chain alkanes and complex PAH in heavy oil. Monosaccharides act as inducers of enzyme activity while providing energy for cell maintenance and proliferation during pollutant biodegradation [14]. To improve the biodegradation efficiency of heavy oil, three organic carbon sources (glucose, sucrose, and soluble starch) were tested as co-substrates for *A. corrugatus* FH2, *A. terreus* FL4, and *Al. alstroemeriae* FW1. Increased colony diameters indicated that the fungal growth was significantly promoted when supplemented with additional carbon sources (*p* < 0.05; Figure 1b). This result is in line with a previous study showing that the degradation of heavy oil sludge by *Purpureocillium lilacinum* and *Penicillium chrysogenumwas* was accelerated in the presence of co-substrates [25].

In terms of colony diameter, FH2 (8.5 mm), FL4 (4.7 mm), and FW1 (10.3 mm) showed limited growth in the control group without co-substrate addition (Figure 1b). The growth of all three fungal strains was significantly enhanced when 0.2% sucrose was added as a co-substrate. FH2 showed the largest colony diameter (48.3 mm), followed by FL4 (46.7 mm) and FW1 (37.0 mm), indicating the pronounced effect of sucrose on stimulating fungal growth and metabolic activity. When 0.2% soluble starch was used, the three strains also exhibited notable increase in growth, with colony diameters reaching 41.7 mm (FH2), 40.3 mm (FL4), and 41.0 mm (FW1). The colony diameters of FH2 and FL4 reached 47.3 mm and 45.3 mm, respectively, when using 0.2% glucose as an auxiliary carbon source. This indicates that glucose possesses great potential for promoting *Aspergillus* growth. However, glucose was a less favorable co-substrate for *Alternaria* growth, as FW1 showed a colony diameter of 35.3 mm, albeit still 3.4-fold higher than that of the control group. Govarthanan et al. [35] isolated a filamentous fungal strain, *Penicillium* sp. CHY-2, from an Antarctic soil, which achieved 49% degradation of decane and 33% degradation of dodecane at 20 °C. The addition of glucose (5.0 g L^−1^) and Tween-80 (5.0 g L^−1^) improved fungal degradation of decane by 1.8- and 1.6-fold, respectively. Collectively, these results demonstrate that sucrose and soluble starch are effective co-substrates strongly promoting the growth and metabolism of fungal strains on heavy oil, which is vital for soil bioremediation. Taking into account the preference of all three fungal strains for co-substrates, sucrose is considered the optimal carbon source. Although glucose can be readily utilized by FH2 and FL4, it potentially induces stress in FW1 under high osmotic pressure, thereby limiting its growth.

In all cases, the fungal growth with heavy oil as the sole carbon source was inferior to that with heavy oil and a small amount of glucose, sucrose, or soluble starch as co-substrate (Figure S1). This confirms that the addition of a co-substrate stimulated the growth performance of the three fungal strains on heavy oil agar plates. It has been reported that adding easily degradable small molecular organics, like glucose and fructose, provides ample carbon nutrients and enhances energy provision. This in turn promotes the biosynthesis and/or secretion of extracellular enzymes, such as CYP monooxygenases, LaC, and peroxidases, which play a role in the oxidation of recalcitrant organic compounds [36]. The co-substrate not only serves as an easily metabolized carbon and nitrogen source to support microbial growth. More importantly, the co-substrate can facilitate the degradation of recalcitrant compounds by inducing dioxygenase activity, as well as enhance cell metabolism by strengthening the antioxidant defense system and accelerating electron transfer efficiency [37]. Therefore, co-metabolic degradation has emerged as a promising solution to the biodegradation of organic compounds, which can enhance the removal efficiency of recalcitrant pollutants from soils. Moreover, yeast extract was added to the MSM containing 40.0 g L^−1^ of heavy oil as an auxiliary nitrogen source. In our previous study, two *Aspergillus* strains (*A. terreus* HJ2 and *A. nidulans* HJ4) showed outstanding growth on extra-heavy oil when supplemented with 3.0 g L^−1^ of yeast extract [27]. The addition of organic nitrogen sources may induce the expression of extracellular enzymes and enhance the degradation rate of petroleum hydrocarbons [38]. Therefore, sucrose and yeast extract were selected as the optimal supplementary carbon and nitrogen sources for *A. corrugatus* FH2, *A. terreus* FL4, and *Al. alstroemeriae* FW1 in subsequent experiments.

### 3.3. Performance of Fungal Consortia with Synergistic Interactions

Biodegradation of heavy oil involves intricate metabolic pathways and enzymatic processes [39]. This necessitates the use of microbial consortia that can degrade heavy oil pollutants via synergistic and metabolic interactions of different members. To achieve higher efficiency and broader spectrum of heavy oil biodegradation, the three fungal isolates affiliated with well-documented oil-degrading genera (*A. corrugatus* FH2, *A. terreus* FL4, and *Al. alstroemeriae* FW1) were combined to develop synthetic consortia. These strains were anticipated to exhibit petroleum hydrocarbon degradation activities and cooperative traits, such as metabolic exchanges and microbial interactions. Four fungal consortia were constructed using a bottom-up design strategy. Cao et al. [16] reported that compared to individual strains, microbial consortia constructed by the bottom-up approach can indeed improve the biodegradation efficiency of complex organic pollutants. Within a consortium, rational division of metabolic pathways can reduce cross-reactions and thus alleviate the metabolic burden on member strains. Our fungal consortia were designated F12 (*A. corrugatus* FH2 + *A. terreus* FL4), F13 (*A. corrugatus* FH2 + *Al. alstroemeriae* FW1), F23 (*A. terreus* FL4 + *Al. alstroemeriae* FW1), and W123 (*A. corrugatus* FH2 + *A. terreus* FL4 + *Al. alstroemeriae* FW1).

**Figure 3:**
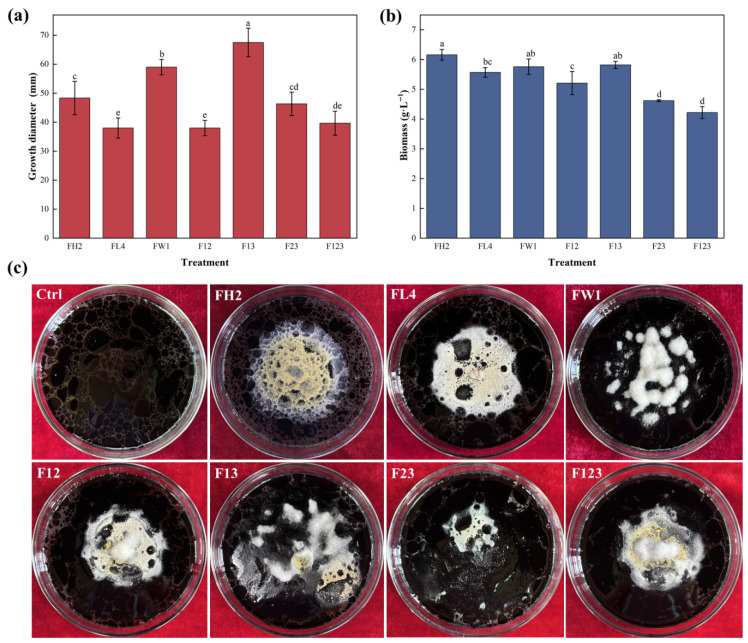
Growth diameter (**a**), cell biomass (**b**), and growth performance (**c**) of three selected fungal strains (FH2, FL4 and FW1) and consortia (F12, F13, F23, F123) in potato dextrose agar medium with heavy oil as a sole carbon and energy source after incubation for 7 days. F12: FH2 + FL4; F13: FH2 + FW1; F23: FL4 + FW1; F123: FH2 + FL4 + FW1. Error bars represent standard deviation of the mean (*n* = 3). Significant differences between groups are indicated by different lowercase letters above the error bars based on Duncan’s multiple range test (*p* < 0.05).

The performance of the four fungal consortia was evaluated based on their cell biomass and growth diameter on the biodegradation medium. The consortium F13 (FH2 + FW1, 1:1, *v*/*v*) exhibited a larger colony diameter (mean 67.5 mm) on heavy oil plates than other single strains and consortia (Figure 3a,c). Two single strains (FW1: 59.0 mm, FH2: 48.3 mm) surpassed the other three consortia (F23: 46.3 mm, F123: 39.7 mm, F12: 38.0 mm) in terms of growth diameter. The consortium F13 showed an improved ability to utilize heavy oil compared to the single strains, which indicates metabolic complementarity between FH2 and FW1. This complementarity possibly involves cross-utilization of secondary metabolites or differentiation of predicted and realized niches for hydrocarbon biodegradation. Chen et al. [40] emphasized that synthetic consortia have better adaptability and greater biodegradation efficiency than pure microbial systems via synergistic and metabolic interactions of individual members. Some strains can break down complex pollutants into small-molecule sugars, which can be used as carbon sources for other strains in the system. Thus, microbial cooperation and co-metabolism (including metabolic exchange) considerably promote biodegradation of long-chain alkanes and PAHs [41]. Notably, the three-strain consortium F123 showed inferior growth (diameter 39.7 mm) compared to F13. This likely suggests that the introduction of *A. terreus* FL4 compromised the synergy of *A. corrugatus* FH2 and *Al. alstroemeriae* FW1 through competition or metabolic redundancy.

Despite their increased growth diameters, the fungal consortia produced less or comparable cell biomass within 5 days than single strains (Figure 3b). The highest values of cell biomass were observed for single strains FH2 (6.2 g L^−1^) and FW1 (5.8 g L^−1^) on oil plates, followed by FL4 (5.6 g L^−1^). The lowest values of cell biomass were recorded for the consortia F23 (4.6 g L^−1^) and F123 (4.2 g L^−1^). The consortium F13 produced moderate cell biomass (5.8 g L^−1^) between FH2 and FW1, exceeding that of other consortia and two single strains (FL4 and FW1). In some cases, a microbial consortium does not exhibit growth advantages compared to single strains due to antagonism or competition between specific members [42]. Based on the growth performance of fungal consortia and single strains on oil plates, the consortium F13 seems to possess diverse metabolic functions through cooperation of its member strains. Thus, F13 (*A. corrugatus* FH2 + *Al. alstroemeriae* FW1) is preliminarily screened out as the optimal fungal consortium for heavy oil biodegradation.

### 3.4. Removal and Biodegradation of Heavy Oil in Simulated Soil

#### 3.4.1. Heavy Oil Removal

**Figure 4:**
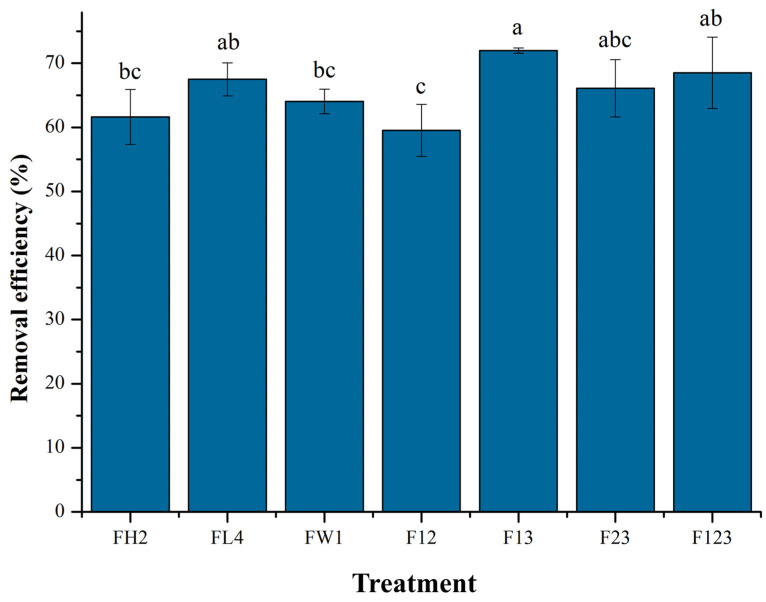
Removal efficiency of heavy oil in simulated soil by fungal strains (FH2, FL4, FW1) and consortia (F12, F13, F23, F123). Error bars indicate standard deviation of the mean (*n =* 3). Different letters above the error bars indicate significant differences between groups (*p* < 0.05) according to Duncan’ s multiple range test.

**Figure 5:**
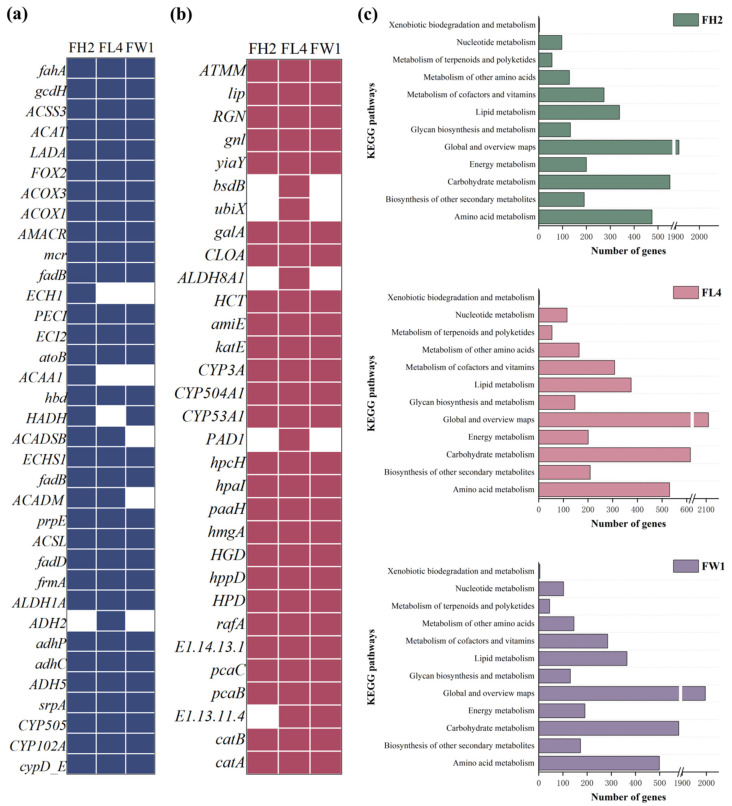
Distribution of core genes for degradation of *n*-alkanes (**a**) and aromatics (**b**), and (**c**) KEGG metabolic pathways identified from the genomes of FH2, FL4, and FW1.

To verify the bioremediation potential of the consortium F13, the four fungal consortia and three single strains were separately inoculated into the bioremediation medium containing heavy oil (2.0%, *v*/*v*) as the primary carbon source and supplemented with sucrose and yeast extract as co-substrates. Thirty days later, the consortium F13 achieved the highest removal efficiency of heavy oil (72.0%) compared to other consortia (F12, F23, F123) and single strains (FL2, FH4, FW1) (Figure 4). The consortia F123 (68.5%) and F23 (66.1%) showed comparable removal efficiency of heavy oil, followed by F12 (59.5%, *p* < 0.05). Among the single strains, FL4 achieved 67.5% removal of heavy oil, close to the efficiency of FH2 and FW1 (61.6% and 64.0%, *p* > 0.05). The consortium F13 (72.0%) showed notably higher removal efficiency than FH2 (61.6%) and FW1 (64.0%), which indicates a synergy between the two strains in bioremediation. It has been noted that microbial cooperation, including cross-feeding and co-metabolism, reinforces the biodegradation of organic pollutants, such as TPHs and PAHs [15,23]. This cooperation hinges heavily on metabolic exchange, such as the degradation intermediates of the targeted pollutants. *A. corrugatus* FH2 and *Al. alstroemeriae* FW1, which served as cooperators in the consortium F13, harbored core genes for degradation of the intermediates of *n*-alkanes and PAHs, such as aliphatic acid, catechol, and protocatechuate (Figure 5). These functional genes likely play a key role in the efficient biodegradation of heavy oil.

The degradation rate of heavy oil by the consortium F12 (59.5%) was lower than that of the single strain FL4 (67.5%). This suggests that *A. corrugatus* FH2 might inhibit the activity or metabolism of *A. terreus* FL4 during the biodegradation process. The three-strain consortium F123 (68.5%) was superior to most single strains and two-strain consortia, but it yielded a 4.0% lower degradation rate than the optimal consortium F13. This provides a hint on potential nutrient competition or metabolic inhibition when the two *Aspergillus* strains (FH2 and FL4) coexisted with the *Alternaria* strain (FW1). Based on the removal efficiency of heavy oil, the consortium F13 (FH2 + FW1) exhibited superiority to other consortia and single strains in heavy oil removal in simulated soil. There are many reports showing that microbial consortia outperform single strains in utilizing heavy oil pollutants as the sole or primary carbon source [43]. Such consortia show an increased degradation rate of heavy oil under laboratory culture conditions. This implies their higher applicability in the bioremediation of soils with complex pollutants, especially heavy oil containing recalcitrant components in complex environments.

Due to its high viscosity, low susceptibility to weathering, and slow degradation, heavy oil is more likely to cause severe toxic pollution than light oil [44]. Thus, efficient biodegradation is fundamental for adequate planning, mitigation, and remediation in the event of heavy oil spills. Microbial consortia present multiple advantages in degrading heavy oil. For instance, the broad substrate spectra, complementary enzymatic activities, and synergistic interactions among consortium members potentially allow them to efficiently utilize and remove heavy oil pollutants [44,45]. Ghorbannezhad et al. [46] found that the sequential fungal–bacterial mixed culture (66.0%) outperformed the fungal–bacterial mixed culture (59.0%), fungal mixed culture (56.6%), and bacterial mixed culture (47.6%) in degrading heavy oil. Our fungal consortium F13 achieved 72.2% removal of heavy oil in simulated soil within 30 days, showing higher biodegradation efficiency than previously reported consortia (32.3% and 66.0%) [47,48]. According to Li et al. [33], when the biodegradation efficiency of crude oil exceeds 50%, microorganisms have already utilized heavy components in crude oil (e.g., aromatics, resins, and asphaltenes) as carbon sources. Thus, the consortium F13 containing *A. corrugatus* FH2 and *Al. alstroemeriae* FW1 has impressive biodegradation capability and bioremediation potential for heavy oil-contaminated soils.

#### 3.4.2. Changes in SARA Fractions

Heavy oil can be separated into four major chemical fractions, i.e., saturates (S), aromatics (A), resins (R), and asphaltenes (A) by chromatographic technique [28]. The SARA profiles of residual oil samples treated with the constructed fungal consortia and selected single strains or not were compared (Table 1). Following 30 days of incubation, 63.6–84.2% of aromatics, 0.7–66.4% of resins, and 9.1–43.0% of asphaltenes in heavy oil were degraded across the fungal treatments. Interestingly, saturates were increased by 27.2–94.2% in heavy oil after treatment with single strains, but not with the consortia. Among them, saturates showed the highest degradation rates (87.4–94.2%), followed by aromatics (85.8–92.0%), resins (66.2–71.4%), and asphaltenes (12.4–28.1%). Under the same conditions, SARA fractions of crude oil differ in their susceptibility to biodegradation or bioconversion due to variations in their polarity and structural complexity [48]. The increased content of saturates in single strain-treated heavy oil is attributed to the degradation of other heavy fractions (aromatics, resins, asphaltenes). Raheem et al. [49] indicated that the biodegradation or bioconversion of heavy oil should lead to increase in the saturates content in parallel with reduction in the aromatics, resins, and asphaltenes contents, as the latter three fractions are more recalcitrant to biodegradation.

For aromatics, the consortia F13 and F123 achieved the degradation rate of 92.0% and 90.1%, respectively, surpassing the most effective single strain FW1 (84.2%). There are reports showing that *Alternaria* species are superior to other fungal strains in utilizing PAHs as the sole carbon and energy source [8,26]. For instance, *Al. tenuissima* 5c-12 isolated from crude oil-contaminated soil in the Yadavaran oil field of southern Iran exhibits potent ability to biodegrade substantial quantities of HMW PAHs, including pyrene and benzo(a)pyrene. This strain achieves remarkable PAH degradation rates ranging from 93.0% to 99.0% [26]. Hence, it is not surprising that the presence of *Al. alstroemeriae* FW1 in the consortia F13 and F123 notably enhanced the biodegradation efficiency of aromatics compared to any individual strains. Aromatics (especially PAHs) in heavy oil are the key components limiting biodegradation [50]. The effective degradation of aromatics by the consortium F13 thus highlights its ability for heavy oil biodegradation. Typically, resins and asphaltenes are considered to be highly resistant to microbial degradation, and this amount of degradation would be accompanied by a decrease in other fractions (saturates and aromatics). Our fungal consortia consisting of *Aspergillus* and *Alternaria* strains enable the biodegradation of resins and asphaltenes to a higher degree (66.2–71.4%) than previously reported (58.2% and none) by Ghorbannezhad et al. [46]. The degradation rate of asphaltene by the consortium F13 was lower (27.3%) than that of *Alternaria* sp. (43.8%) isolated from deep-sea sediment in the Gulf of Mexico [8]. However, the efficient degradation of resins within 30 days observed in this study is promising. Given their complex polyaromatic structure and low bioavailability, resins and asphaltenes are widely recognized as the most recalcitrant fractions of crude oil. Developing microbial consortia targeted to specific pollutants and environmental conditions could enhance the degradation efficiency. Overall, the consortium F13 shows superior biodegradation performance for aromatics, resins, and asphaltenes, which renders it favorable for practical application in soil bioremediation. Daccò et al. [9] highlighted the importance of white-rot fungi (e.g., *Pleurotus* and *Trametes*) for PAH biodegradation due to their unique enzyme systems. In future work, we will therefore screen for white-rot fungi from multiple sample sets to construct novel microbial consortia, combining these with the ascomycetous isolates (*A. corrugatus* FH2 and *Al. alstroemeriae* FW1) used in this study to target the degradation of macromolecules such as resins and asphaltenes.

#### 3.4.3. *n*-Alkane Biodegradation

The effectiveness of the fungal consortia and single strains in biodegrading *n*-alkanes was verified through GC–MS analysis. All strains (FH2, FL4, FW1) and consortia (F12, F13, F23, F123) were able to utilize a wide range of *n*-alkanes between C_13_–C_28_ (Table 2). Treatment with the consortia highly promoted the degradation of *n*-alkanes with different carbon numbers compared to single strains, especially for long-chain alkanes (C_24_–C_28_). This is exemplified by the notably higher mean degradation rate of C_24_–C_28_ by the consortium F13 (95.9%) than that of *A. corrugatus* FH2 (49.7%), *A. terreus* FL4 (28.8%), and *Al. alstroemeriae* FW1 (68.6%). The enhanced ability of mixed cultures to degrade petroleum hydrocarbon pollutants can be attributed to the synergies of multiple strains [45].

Fungal consortia provide a richer mix of enzymes relative to single strains, allowing them to achieve higher biodegradation efficiency. F12 achieved 64.2% degradation of C_13_–C_28_ in total. With respect to medium-chain alkanes (C_13_–C_16_), the highest degradation rate in F12 treatment was recorded for C_16_ (96.1%) and the lowest for C_13_ (49.7%), alongside a noticeable increase in the relative abundances of C_15_ (134.5%). Notably, F12 showed greater degradation capability for long-chain alkanes (C_17_–C_28_, mean 65.5%) than for medium-chain alkanes (C_13_–C_16_, mean 17.4%). In particular, 94.4% of tetracosane, 76.9% of pentacosane, and 100.0% of heptacosane were degraded after treatment with F12, exceeding the maximum rates observed with single strains (76.1%, 52.8%, 78.7%). F13 achieved 76.0% degradation of C_13_–C_28_ in total. This consortium also showed a greater propensity for degrading long-chain alkanes (C_17_–C_28_, mean 80.8%) than medium-chain alkanes (C_13_–C_16_, mean 61.8%), as exemplified by tetracosane (97.7%), pentacosane (97.0%), and heptacosane (91.5%). Strain FW1 exhibited comparable capability to degrade medium-chain alkanes (C_13_–C_16_, mean 39.4%) and long-chain alkanes (C_24_–C_28_, mean 68.6%). The addition of FW1 to the consortium F13 enhanced the mean degradation rate of long-chain alkanes (C_24_–C_28_) to 95.9%. These quantitative results demonstrate the synergies of fungal strains in a consortium, which foster biodegradation of more complex hydrocarbons in heavy oil.

In the F23 treatment, both medium-chain and long-chain alkanes decreased in relative abundance, with the mean degradation rate reaching 90.8% (C_13_–C_16_) and 84.1% (C_17_–C_28_). Notably, 90.9% of tetracosane, 91.4% of pentacosane, and 100.0% of heptacosane were degraded after F23 treatment. F123 only achieved 32.1% degradation of medium-chain alkanes (C_13_–C_16_) on average, with the highest rate for C_13_ (93.2%) and the lowest for C_16_ (87.6%). This was accompanied by a large increase in the relative abundance of C_15_ (144.7%). However, F123 was effective at degrading long-chain alkanes (C_17_–C_28_, mean 78.5%), including tetracosane (94.5%), pentacosane (76.1%), and heptacosane (96.4%), which surpassed single strains (76.1%, 52.8%, 78.7%). The results indicate that by combining the *Aspergillus* and *Alternaria* strains with petroleum hydrocarbon biodegradation capabilities, their degradation efficiencies for long-chain alkanes in heavy oil are substantially improved. Degradation of long-chain alkane by microbial consortia through multiple metabolic mechanisms is a promising strategy for remediating contaminated areas impacted by persistent pollutants, such as petroleum hydrocarbons.

Compared to other microbial consortia reported previously, the fungal consortium F13 has distinct advantage in degrading heavy oil and its *n*-alkanes components (Figure 4 and Table 2). Dai et al. [51] developed a bacterial consortium using *Brevibacillus* sp. DL-1, *Bacillus* sp. DL-13, and *Acinetobacter* sp. DL-34, which achieved 39.4% degradation of heavy oil within 100 days. Medaura et al. [13] constructed a consortium with six potentially hydrocarbonoclastic fungi of the genera *Penicillium*, *Ulocladium*, *Aspergillus*, and *Fusarium*, which showed 39.9% degradation of TPHs (C_14_–C_35_) after 120 days of incubation. These bacterial and fungal consortia are inferior to F13 (72.0% in 30 days), underscoring the ability of this optimal consortium to utilize *n*-alkanes. To data, many microbial consortia have been used to degrade petroleum hydrocarbons, where different species undertake various functions in the biodegradation process. This can reduce the growth pressure of individual strains and enhance their tolerance to harsh environments through co-metabolism or cooperation (e.g., “hitchhiking” strategy), which renders the biodegradation system more stable and robust [16,23]. Taking into account its remarkable biodegradation activity for heavy oil and long-chain alkanes, the fungal consortium F13 has excellent potential for bioremediation of heavy oil-contaminated soils.

### 3.5. Whole-Genome Sequencing of Heavy Oil-Degrading Fungi

#### 3.5.1. Genomic Features of Fungal Strains

The general genomic features of the three fungal strains used to develop heavy oil-degrading consortia are provided in Table 3 and Figure S2. The total length of the fungal genomes was 30,458,146 base pairs (bp) for FH2, 32,757,900 bp for FL4, and 34,353,871 bp for FW1. The number of contigs was 8 (FH2), 12 (FL4), and 11 (FW1), with the average GC content of 50.0% (FH2), 52.0% (FL4), and 51.0% (FW1). The genome of FH2 consisted of 9355 coding sequences (CDSs), 220 tRNA genes, and 44 rRNA genes (18S: 4, 5.8S: 4, 5S: 36). The genome of FL4 harbored 10,459 CDSs, 169 tRNA genes, and 64 rRNA genes (18S: 10, 5.8S: 20, 5S: 34). The genome of FW1 contained 12,749 CDSs, 124 tRNA genes, and 74 rRNA genes (18S: 14, 5.8S: 17, 5S: 43). Genome annotation of FH2, FL4, and FW1 was performed using BLAST with open-access databases, NR (9141, 10,177, 12,546 genes), GO (7578, 7966, and 8316 genes), KOG (1776, 7359, and 1897 genes), and KEGG (6760, 7359, and 7333 genes) (Table 3). The GO analysis revealed that 3.5–4.4% of the genes were related to oxidoreductase activity, 1.8–2.2% were responsible for monooxygenase activity, 2.1–2.8% participated in hydrolase activity, and 3.1–3.9% were involved in transmembrane transporter activity. Numerous genes were associated with KEGG metabolic pathways, including the metabolism of cofactors and vitamins, lipid metabolism, carbohydrate metabolism, amino acid metabolism, and biosynthesis of other secondary metabolites (Figure 5c). The diversity of functional annotations provides genetic evidence for our fungal strains to degrade petroleum hydrocarbons in heavy oil.

The KEGG annotation showed that among the three fungal strains, more than 4000 metabolism-related genes were mapped to 12 metabolic pathways (Figure 5c). Specifically, 6760 (72.3%), 7359 (70.4%), and 7333 (57.5%) of the CDSs were, respectively, identified in 12 KEGG pathways for FH2, FL4, and FW1. These pathways included xenobiotic biodegradation and metabolism (3, 3, 5), amino acid metabolism (475, 531, 499), carbohydrate metabolism (550, 615, 579), lipid metabolism (339, 375, 365), metabolism of cofactors and vitamins (274, 308, 285), and energy metabolism (200, 201, 191). The results suggest that a large number of annotated genes present in the three genomes perform functions via essential pathways of primary metabolism, which can guarantee fungal growth on heavy oil. Comprehensive genome annotation of the three strains revealed their metabolically versatile genomes enriched with genes related to the pathways for oxidative degradation and xenobiotic biodegradation and metabolism, such as hydrocarbon metabolism. Among the functional genes participated in hydrocarbon metabolism, there were 34 (FH2), 32 (FL4) and 30 (FW1) core genes involved in *n*-alkane degradation, 30 of which were shared among the three strains. Additionally, 24 (FH2), 29 (FL4), and 25 (FL4) core genes were involved in aromatic degradation, 24 of which were shared among the three strains (Figure 5a,b).

#### 3.5.2. Functional Genes Responsible for Heavy Oil Biodegradation

Identification of functional genes that encode hydrocarbon-degrading enzymes is crucial for uncovering the potential of heavy oil biodegradation by microbial consortia. This also provides molecular insight into the metabolic mechanisms of biodegradation and gene interactions involved in complex biodegradation pathways [52]. Herein, the distribution of core genes for degradation of *n*-alkanes and aromatics in fungal strains FH2, FL4, and FW1 were analyzed. Figure 5a shows the core genes involved in *n*-alkane degradation, including *CYP*, *ADH5*, *frmA*, *gcdH*, and *fadD* that participate in *n*-alkane catabolism. The *CYP* genes (*CYP102A*, *CYP505*, *cypD_E*) have been suggested to play an indispensable role in *n*-alkane degradation. These genes encode CYP monooxygenases with redox and hydroxylation activities, which catalyze the oxidation of terminal C-H groups in *n*-alkanes to form alcohols and are widely used in long-chain alkane degradation [53]. Daccò et al. [9] demonstrated that fungal degradation of *n*-alkanes is commonly attributed to the action of enzymes in the CYP families. Filamentous fungi tend to possess more CYP genes, and the CYP families consist of distinct CYP forms that may contribute as a set of isoforms to the metabolic conversion of a substrate. Additionally, CYP monooxygenases mediate diverse biological processes (adaptation to environmental stresses and new niches, metabolization of endogenous and xenobiotic compounds), directly contributing to fungal fitness [54].

The *ADH5*, *frmA, adhC*, and *adhP* genes encode alcohol dehydrogenases, which act as biocatalysts for the conversion of primary alcohols into aldehyde during long-chain alkanes catabolism. The *ALDH1A* and *fahA* genes encode aldehyde dehydrogenases, which oxidize aldehyde into fatty acids. Next, β-oxidation of fatty acids is catalyzed through transferase and enoyl-CoA hydratase encoded by *atoB*, *fadB*, *fadD*, and *paaH*. Regarding fatty acid metabolism, all three fungal strains carried a complete set of functional genes related to β-oxidation, including *ACOX* for acyl-CoA oxidase), *ACSL* and *fadD* for acyl-CoA synthases, *fadB* and *ECHS1* for acyl-CoA hydratases, *HADH* for hydroxyacyl-CoA dehydrogenase, and *ACAT* and *atoB* for thioesterases. The presence of these genes enables the fungal strains to achieve oxidative degradation of *n*-alkanes. Li et al. [55] reported that the gene cluster *ACOX* catalyzes the first step of the fatty acid β-oxidation pathway and plays a crucial role in the later stage of hydrocarbon biodegradation. Notably, the three fungal strains harbored genes like *AMACR* and *mcr* that encode enzymes for breakdown of branched-chain alkanes, indicating their potential to degrade complex components in crude oil. However, FL4 lacked the *HADH* gene, potentially impairing its hydroxyacyl-CoA dehydrogenase activity, whereas both FH2 and FW1 possessed a more complete β-oxidase system. The genomes of FH2 and FW1 could encode ferredoxin and ferredoxin reductase, enabling the transfer of electrons from HADH to CYP and thereby aiding *n*-alkane oxidation. Further, the presence of multiple acyl-CoA oxidase genes (*ACOX1* and *ACOX3*) enhances the capacity of the fungal strains to oxidize fatty acids. The *CYP* genes in FW1 could optimize electron transfer efficiency within the CYP oxidation system, while relying on *ACAA1* and *ECH* to enhance the terminal step of β-oxidation. Furthermore, these fungal strains contained other oxygenase genes, such as *gcdH*, *hbd*, *prpE*, and *cyp D_E*, which play a key role in alkane degradation. In particular, *cyp D_E* encodes CYP monooxygenase involved in the degradation of *n*-alkanes and aromatics [52].

Three fungal strains contained multiple core genes for degradation of aromatics, such as *catA*, *catB*, *katE*, *hmgA*, *pcaB*, *pcaC*, and *CYP* (Figure 5b). These catabolic genes encode PAH-degrading enzymes, which aid in the mineralization process. The aerobic degradation of aromatics involves three stages: (i) initial attack or activation, (ii) dearomatization or ring cleavage, and (iii) further degradation through downstream pathways [56]. The initial attack or activation can be performed by mono- or dioxygenases enzymes. The *CYP* genes (*CYP504A1*, *CYP 505*, *cyp D_E*) identified in our fungal strains are responsible for the first reaction. These genes encode CYP monooxygenases to oxidize hydrocarbons and then convert aromatics into hydroxy, dihydroxy, dihydrodiol, and quinone assistants, which are then transferred and reduced by transferases and quinone reductase. This leads to the formation of diol intermediates, such as catechol or protocatechuate, which are further degraded by intra-diol (ortho) or extra-diol (meta) ring cleavage mechanisms [57]. Both FH2 and FW1 harbored the genes *catA*, *catB*, *hmgA*, and *pcaC* encoding catechol dioxygenase, gentisate dioxygenase, and protocatechuate dioxygenase, which help cleave catechol and protocatechuate at the ortho or meta positions [50]. Zhou et al. [58] reported that *pcaC*, *paaH*, and *frmA* are well-known genes involved in the degradation of aromatics. Moreover, FH2, FL4 and FW1 contained a lignin peroxidase-encoding gene (*LiP*), which mediates the degradation of catechol and protocatechuate [59]. The ortho-cleavage pathway produces acetyl-CoA and butanedioic acid, whereas the meta-cleavage pathway generates pyruvate and acetaldehyde, which then enter the tricarboxylic acid (TCA) cycle for complete mineralization.

Some fungi, such as *Phanerochaete chrysosporium*, *Ganoderma lucidum*, and *Trametes maxima*, secrete extracellular peroxidases (e.g., LiP, MnP) that enable oxidative coupling of various PAHs [60]. This catalysis involves the reduction of hydrogen peroxide (H_2_O_2_) as a co-metabolic substrate, along with the oxidation of aromatics. The presence of elevated LiP and MnP activities during the degradation of aromatics (e.g., phenanthrene, pyrene, and benzo(a)pyrene) indicates that these enzymes act as primary players in the biodegradation of selected hydrocarbons [61]. Moreover, some fungi secrete various oxygenases, including monooxygenases or dioxygenases, peroxidase, dehydrogenase, and transferases, facilitating the degradation of aromatics [60]. In earlier degradation stage, aromatic ring oxidation is catalyzed by either monooxygenase or dioxygenase and followed by hydroxylation to form hydrodiol or dihydrodiol intermediates. The aromatic ring is then rearomatized by a dehydrogenase and cleaved to form a catechol intermediate, and ultimately entering the TCA cycle [60,62]. Therefore, fungal clean-up of PAH pollutants is more promising than bacterial treatment. Our fungal strains FH2, FL4, and FW1 contained the *LiP* gene for lignin peroxidase, which mediates enhanced degradation of aromatic compounds. The genes associated with the uptake of medium- and long-chain alkanes and aromatics play a pivotal role in bioremediation of petroleum-contaminated soils. These genes enable efficient assimilation of readily bioavailable hydrocarbons, supporting sustained metabolic activity and energy production. This, in turn, facilitates the degradation of more complex and recalcitrant petroleum hydrocarbons by ensuring microbial energy requirements are met throughout the biodegradation process.

#### 3.5.3. Possible Metabolic Pathways for Petroleum Hydrocarbon Biodegradation

**Figure 6:**
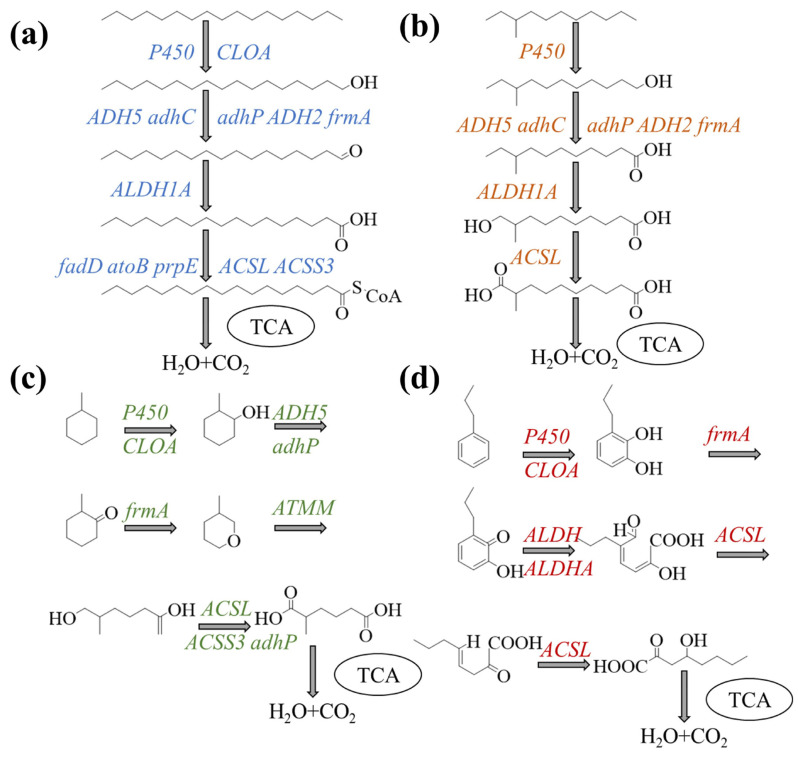
Predicted biodegradation pathways of *n*-heptadecane (**a**), 2-methyl-undecane (**b**), methyl-cyclohexane (**c**), and propyl-benzene (**d**) by fungal strains (FH2, FL4, FW1) based on whole-genome sequencing and hydrocarbon metabolism outcomes. TCA, tricarboxylic acid cycle.

To elucidate the biodegradation of *n*-alkanes and aromatics by the consortium F13, the possible metabolic pathways were reconstructed based on the genome annotation results and hydrocarbon metabolism outcomes (Figure 6). The key enzymes (genes) involved in *n*-alkane degradation include CYP enzymes (*CYP102A*, *CYP505*, *cypD_E*), alcohol dehydrogenase (*ADH5*, *frmA*, *adhC*, *adhP*), aldehyde dehydrogenase (*fahA*, *ALDH*), and β-hydroxylase (*fadB*, *fadD*, *atoB*). The aerobic degradation of *n*-alkanes occurs most frequently through terminal oxidation, sub-terminal oxidation, ω-oxidation, and β-oxidation. This leads to the production of alcohols, which are subsequently dehydroxylated to fatty acids [9]. Tang et al. [52] reported that the first gene responsive for this process varies with substrate. Our results (Figure 6a) indicated that the fungal degradation of linear alkanes started from mono-terminal oxidation. Specifically, linear hydrocarbons were first oxidized to alcohols through CYP enzymes encoded by *CYP505*, *CYP102A*, and *cypD_E* genes, and then converted into aldehydes through alcohol dehydrogenases encoded by *ADH5*, *adhC*, and *adhP* genes. Next, aldehydes were dehydroxylated to fatty acids through enzymes encoded by *fahA* and *ALDH* genes. Finally, fatty acids were metabolized via the β-oxidation pathway, and enzymes encoded by *fadB*, *fadD* and *ACADM* genes catalyzed the decomposition of fatty acids into acetyl-CoA, which subsequently entered the TCA cycle.

Branched-chain alkanes and cycloalkanes were oxidized by fungi via different routes, yielding epoxides, alcohols, diols, and carboxylic acid units (Figure 6b,c). For branched-chain alkanes, one side was first oxidized to alcohols through an enzyme encoded by the gene *cypD_E*, subsequently oxidized to aldehyde through enzymes encoded by the genes *adhC* and *adhP*, and then dehydroxylated to fatty acids; the other side was oxidized to fatty acids (Figure 6b). Chen et al. [63] pointed out that structurally stable branched-chain alkanes are generally degraded via double-end oxidation or β-oxidation. During degradation, cycloalkanes were first converted into cyclic alcohols and then dehydrogenated to ketones by an oxidase system (Figure 6c). Eight core genes were involved in this process, *P450*, *CLOA*, *ADH5*, *adhP*, *frmA*, *ATMM*, *ACSL*, and *ACSS3*. Abbasian et al. [64] suggested that the metabolism of cycloalkanes requires a mono-oxygenase system followed by lactonates ring, and the ring is finally opened by a lactone hydrolase. Two oxygenase systems are almost never found in the same microorganism, making it difficult to degrade cycloalkanes by pure cultures. The fungal strains FH2 and FW1 contained core genes involved in the degradation of *n*-alkanes, alleviating the degradation pressure on single strains and thereby enabling more efficient biodegradation.

Compared to *n*-alkanes, aromatics were degraded by fungi through more complex and variable pathways. Briefly, cyclic hydrocarbons underwent ring hydroxylation by oxidoreductases including mono-oxygenases, followed by ring-opening cleavage at the ortho or meta positions by catechol or gentisate dioxygenases, respectively. These processes yielded succinate, pyruvate, and acetyl-CoA, which were mineralized via the TCA cycle [23]. The core genes involved in the degradation of aromatics were summarized in Figure 6d. The initial attack of aromatics by fungi was assured by CYP monooxygenases (*CYP504A1*, *CYP53A1*, and *cyp D_E*), which incorporated an oxygen molecule into the aromatic ring to form an epoxide. The product subsequently underwent spontaneous isomerization to form diol intermediates, such as catechol or protocatechuate. Jiang et al. [65] have demonstrated that CYP-mediated hydroxylation leads to epoxide-to-diol conversion, supporting our proposed degradation pathway. Then, catechol dioxygenase (*catB*), gentisate dioxygenase (*hmgA*), and protocatechuate dioxygenase (*pcaC*) cleaved catechol and protocatechuate at the ortho or meta positions. The intermediates were converted, finally entering the TCA cycle. Our fungal strains contained the genes *catB*, *hmgA*, and *pcaC*, which could enable efficient biodegradation by alleviating the degradation pressure on single strains. Through functional collaboration of FH2, FL4, and FW1, the range of metabolic spectrum was expanded in fungal consortia (e.g., F13), allowing them to rapidly degrade intermediates from each other. This microbial cooperation promoted avoided heavy oil biodegradation by avoiding product accumulation, which implies the strong potential of fungal consortia for soil bioremediation.

This study suffers from several limitations. First, the potential applicability of the consortium F13 as a reliable fungal resource for soil bioremediation was evaluated based on simulated tests, and the gene function was analyzed at the laboratory scale. In some cases, fluctuating environmental conditions may compromise the degradation and removal efficiency of heavy oil and the stability of microbial degraders. Hence, future research involving field trials is needed to verify the adaptability and stability of the consortium F13 under real-world environmental fluctuations typical of contaminated field sites (e.g., temperature shifts, nutrient limitations). Second, compared to other microbial consortia reported previously, the consortium F13 has distinct advantage in degrading heavy oil and achieves 72.0% of heavy oil degradation. However, the degradation rate of asphaltenes (27.3%) by F13 is inferior to other bacterial consortia. For example, Kshirsagar et al. [66] constructed a bacterial consortium using asphaltene-degrading *P. putida*, *P. mendocina*, *B. cereus*, *B. marisflavi*, *Lysinibacillus fusiformis*, and *Ochrobactrum intermedium.* This six-member consortium was found to degrade asphaltenes by 35.0% under aerobic conditions. The consortium F13 could be optimized further through metabolic engineering, nutrient supplementation, or adaptive evolution by gradually increasing exposure to recalcitrant asphaltene fractions. Genetic engineering and synthetic biology may increase the adaptability of microorganisms, enabling them to target a broader range of contaminants and enhance the feasibility and effectiveness of bioremediation [67]. Third, the metabolic pathways of petroleum hydrocarbon by the consortium F13 are proposed based on genomic annotation results. Despite the consistency with phenotypic data, these pathways should be considered predictive and require validation for genome function relationships. The integration of multi-omics approaches (e.g., transcriptomics, proteomics, metabolomics) can be helpful to uncover degradation genes actively expressed during heavy oil biodegradation and validate pathway activation. Omics data would provide deeper insights into microbial degradation pathways and improve bioremediation strategies. Exploring the catabolic potential of microbes, genes, and structure–unction correlations in hydrocarbon-degrading microbial communities will pave the way for the development of microbial consortia or genetically engineered strains in an ecologically sustainable yet biologically safe manner [60]. Despite challenges like environmental fluctuations and microbial interactions, ongoing research continues to refine bioremediation techniques.

## 4. Conclusions

Three fungal strains with impressive biodegradation efficiency for heavy oil were isolated from oil sludge, designated *Aspergillus corrugatus* FH2, *Aspergillus terreus* FL4, and *Alternaria astroemeriae* FW1. Among the single strains and their consortia, the combination of FH2 and FW1 achieved the highest removal efficiency (72.0%) for heavy oil in simulated soil, indicating the synergy of *Aspergillus* and *Alternaria* strains in biodegradation. The remarkable biodegradation capabilities of these fungal strains were linked to the presence of specific genes in their genomes. For instance, *ADH5*, *frmA*, *adhC*, *fadB*, and *CYP505* genes were responsible for the degradation of *n*-alkanes, and *cypD_E*, *catB*, *katE*, *hmgA*, *ALDH*, and *ACSL* genes were involved in the degradation of aromatics. These functional genes participated in cascades of reactions related to the degradation and transformation of macromolecular components in heavy oil, such as *n*-alkanes, aromatics, resins, and asphaltenes.

This study successfully obtained an efficient heavy oil-degrading fungal consortium, providing a green solution to tackle complex petroleum hydrocarbon pollution and offering reliable strain resources for soil bioremediation. Both strains in the consortium (FH2 and FW1) are recovered from a specific geographical location in Xinjiang with a temperate continental desert/semi-desert climate. These strains are presumably adapted to similar temperate (or mesophilic) conditions within the temperature range of 20–37 °C. We do not expect them to be equally effective in extreme climatic zones without prior adaptation. However, the genera *Aspergillus* and *Alternaria* are known for wide distribution, suggesting their natural plasticity that could be harnessed through further acclimatization. Moreover, our conclusions are based on optimal laboratory conditions, and further verification through field tests is required. Introducing a high-density exogenous fungal consortium carries the inherent risk of disrupting the native soil biocenosis and impacting the local flora and fauna. While this study is primarily focused on fungal degradation efficiency, responsible bioremediation must take into account ecological balance. The use of native strains (e.g., FH2 and FW1 isolated from the contaminated site) partially mitigates this risk, as these fungi are already part of the local gene pool. Future research is needed to verify the stability of the consortium F13 in real contaminated sites. Targeted interventions can be designed through metabolic pathway regulation in order to promote the practical application of microbial-mediated remediation of soils.

## Figures and Tables

**Table 1:** Degradation rates of saturates, aromatics, resins, and asphaltenes by fungal strains (FH2, FL4, FW1) and consortia (F12, F13, F23, F123) in simulated soil bioremediation test.

Treatment	Saturates (mg g^−1^)	VR_m_%	Aromatics (mg g^−1^)	VR_m_%	Resins (mg g^−1^)	VR_m_%	Asphaltenes (mg g^−1^)	VR_m_%
Ctrl	103 ± 9 d	–	690 ± 19 a	–	420 ± 16 a	–	121 ± 5 a	–
FH2	179 ± 17 b	−73.8	251 ± 8 b	63.6	417 ± 33 a	0.7	82 ± 4 bc	32.2
FL4	131 ± 4 c	−27.2	135 ± 5 c	80.4	141 ± 12 c	66.4	110 ± 8 a	9.1
FW1	200 ± 2 a	−94.2	98 ± 1 d	84.2	189 ± 4 b	55.0	69 ± 13 c	43.0
F12	9 ± 1 e	91.3	84 ± 13 de	85.8	142 ± 1 c	66.2	87 ± 9 b	28.1
F13	13 ± 1 e	87.4	55 ± 3 f	92.0	128 ± 7 c	69.5	88 ± 2 b	27.3
F23	8 ± 3 e	92.2	78 ± 5 de	88.7	135 ± 27 c	67.9	106 ± 2 a	12.4
F123	6 ± 1 e	94.2	68 ± 1 ef	90.1	120 ± 18 c	71.4	106 ± 14 a	12.4

Values are presented as the mean ± standard deviation (*n* = 3). Different letters within the same column indicate significant differences between groups (*p* < 0.05) according to Duncan’s multiple range test. VR_m_% represents the degradation rate in a fungal treatment group relative to the control group.

**Table 2:** Changes in the relative abundance of *n*-alkanes in heavy oil after biodegradation by fungal strains (FH2, FL4, FW1) and consortia (F12, F13, F23, F123) based on gas chromatography–mass spectrometry.

Retention Time (s)	Molecular Formula	Relative Change (%)						
		FH2	FL4	FW1	F12	F13	F23	F123
1850.9	C_13_H_28_	66.1	140.5	43.0	−49.7	−89.5	−98.3	−93.2
1988	C_14_H_30_	−94.9	−48.1	−59.9	−58.5	−68.2	−90.8	−92.2
2118.3	C_15_H_32_	−38.4	110.9	−48.6	134.5	−36.3	−83.7	144.7
2242.1	C_16_H_34_	−36.9	−89.9	−91.9	−96.1	−53.3	−90.5	−87.6
2360.1	C_17_H_36_	−23.8	−26.5	−54.3	−16.5	−47.9	−56.8	−76.5
2472.6	C_18_H_38_	−22.6	22.7	−14.5	−68.9	−74.8	−82.2	−73.7
2580.4	C_19_H_40_	−96.2	136.8	64.2	−98.2	−98.2	−89.3	−61.6
2683.7	C_20_H_42_	−14.6	171.6	11.2	−86.2	−99.0	−92.6	−85.0
2782.6	C_21_H_44_	−92.4	67.1	−94.6	−89.6	−92.3	−96.6	−90.4
2877.4	C_22_H_46_	−89.3	52.7	55.0	0.8	−25.6	−74.2	−15.6
2968.6	C_23_H_48_	−92.1	−83.7	−16.7	36.1	−51.9	−53.5	−79.0
3056.4	C_24_H_50_	−76.1	−47.3	−72.7	−94.4	−97.7	−90.9	−94.5
3146.2	C_25_H_52_	−0.4	35.1	−52.8	−76.9	−97.1	−91.4	−76.1
3246.3	C_26_H_54_	−71.0	−96.8	−87.4	−91.9	−93.3	−81.5	−93.1
3360.1	C_27_H_56_	−71.2	51.3	−78.7	−100.0	−91.5	−100.0	−96.4
3499.8	C_28_H_58_	−29.8	33.5	−51.4	−100.0	−100.0	−100.0	−100.0

**Table 3:** Genomic features of three heavy oil-degrading fungal strains.

Feature	Description		
	*Aspergillus corrugatus* FH2	*Aspergillus terreus* FL4	*Alternaria alstroemeriae* FW1
Genome size (bp)	30,458,146	32,757,900	34,353,871
Genome coverage	619.28×	503.64×	555.79×
Completeness	99.80	99.70	99.80
No. of contigs	8	12	11
G + C content (%)	49.97	52.04	51.04
*N*_50_	4,088,792	4,299,909	3,087,319
*N*_90_	3,190,075	2,050,326	2,351,359
Maximum scaffold size (bp)	8,265,442	5,300,274	7,341,161
Median scaffold size (bp)	3,435,058	3,360,821	2,599,701
Minimum scaffold size (bp)	141,407	48,728	50,633
Average scaffold size (bp)	3,435,058	2,729,825	3,123,079
Protein-coding genes	9355	10,459	12,749
Average mRNA size (bp)	2888	2473	2242
No. of exons	31,736	33,842	33,949
No. of introns	22,381	23,383	21,200
tRNAs	220	169	124
rRNAs	4, 0, 4, 36 (18S, 28S, 5.8S, 5S)	10, 0, 20, 34 (18S, 28S, 5.8S, 5S)	14, 0, 17, 43 (18S, 28S, 5.8S, 5S)
Small nuclear RNAs	33	30	37
Genes assigned to NR	9141	10,177	12,546
Genes assigned to GO	7578	7966	8316
Genes assigned to KEGG	6760	7359	7333
Genes assigned to KOG	1776	7359	1897

## Data Availability

The original contributions presented in this study are included in the article/supplementary material. Further inquiries can be directed to the corresponding author.

## Supplementary Materials

The following supporting information can be downloaded at: https://www.mdpi.com/article/10.3390/jof12030224/s1, Figure S1: Growth performance of three selected fungal strains (FH2, FL4, FW1) on the basal mineral salts medium containing heavy oil as the sole carbon source (C) and supplemented with 0.2% glucose (G), soluble starch (So), or sucrose (Su) as an additional carbon source (all incubated for 5 days); Figure S2: Circular visualization of the complete genomes of three efficient heavy oil-degrading fungi isolated from oil sludge, *Aspergillus corrugatus* FH2 (a), *Aspergillus terreus* FL4 (b), and *Alternaria alstroemeriae* FW1 (c). For the circular chromosomes, circles from the outside to the center indicate: chromosome coordinates, chromosome names, gene density (calculated in 50 kbp windows), repeat sequence density, GC content, and synteny blocks.
