# Supplementary material for: Development and Assessment of Heavy Oil-Degrading Fungal Consortia (Aspergillus and Alternaria) for Soil Bioremediation
Source: J Fungi (Basel). 2026 Mar 19;12(3):224. doi: 10.3390/jof12030224 (PMC13027517; doi:10.3390/jof12030224)
Supplement: Supplementary file 1 [file jof-12-00224-s001.zip › jof-4161659-supplementary.pdf]

Supplementary file for

Development and assessment of heavy oil-degrading fungal consortia (*Aspergillus* and *Alternaria*) for soil bioremediation

Shujuan Peng, Junhao Zhu, Weiguo Liu, Junhui Zhang*

College of Ecology and Environment, Key Laboratory of Oasis Ecology of the Ministry of Education, Xinjiang University, Urumqi 830046, China

* Correspondence: Junhui Zhang

Postal address: College of Ecology and Environment, Xinjiang University, No. 777 Huarui Street, Urumqi 830046, China

E-mail address: zhangjunhui6475@xju.edu.cn (J. Zhang)

Phone: +86-991-8582337

Fax: +86-991-8582337

FIGURES

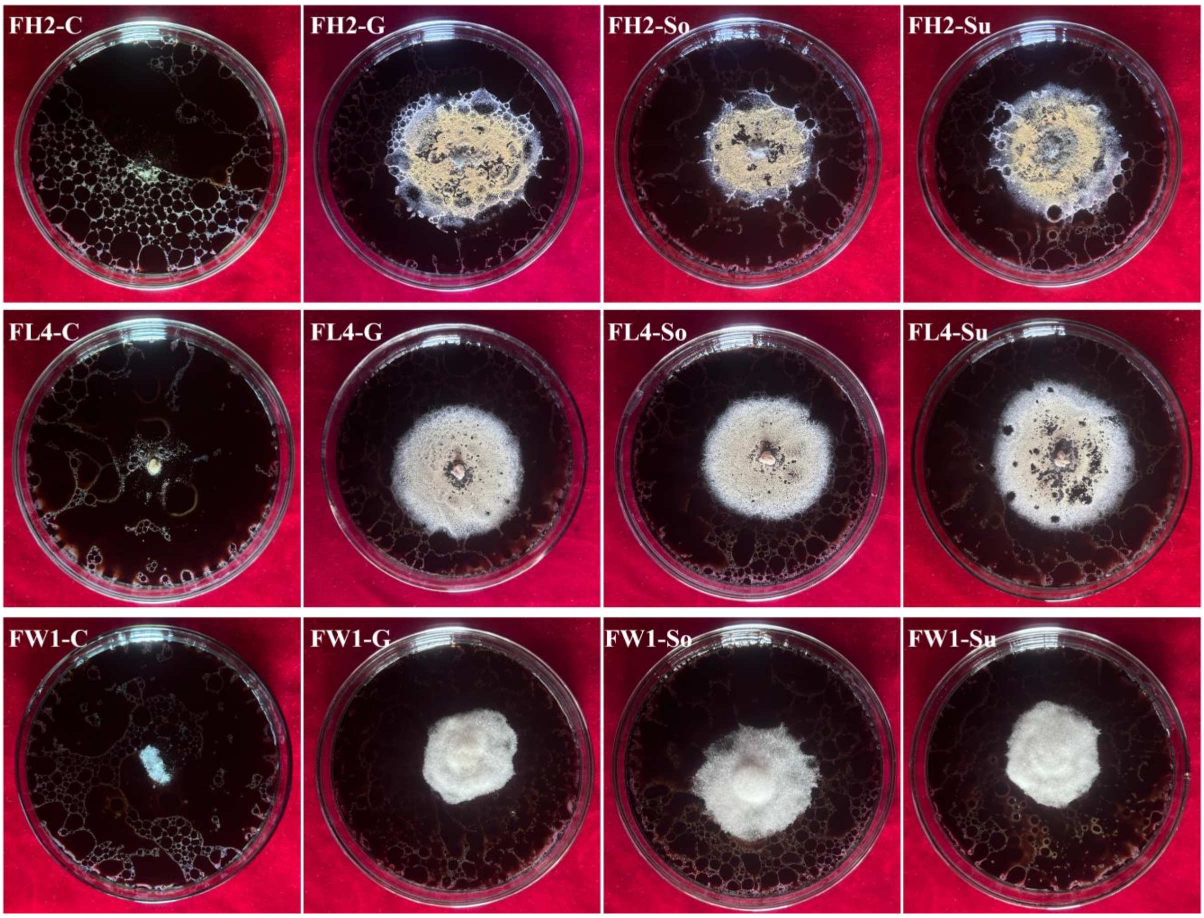

Figure S1. Growth performance of three selected fungal strains (FH2, FL4, FW1) on the basal mineral salts medium containing heavy oil as the sole carbon source (C) and supplemented with 0.2% glucose (G), soluble starch (So), or sucrose (Su) as an additional carbon source (all incubated for 5 days).

(a)

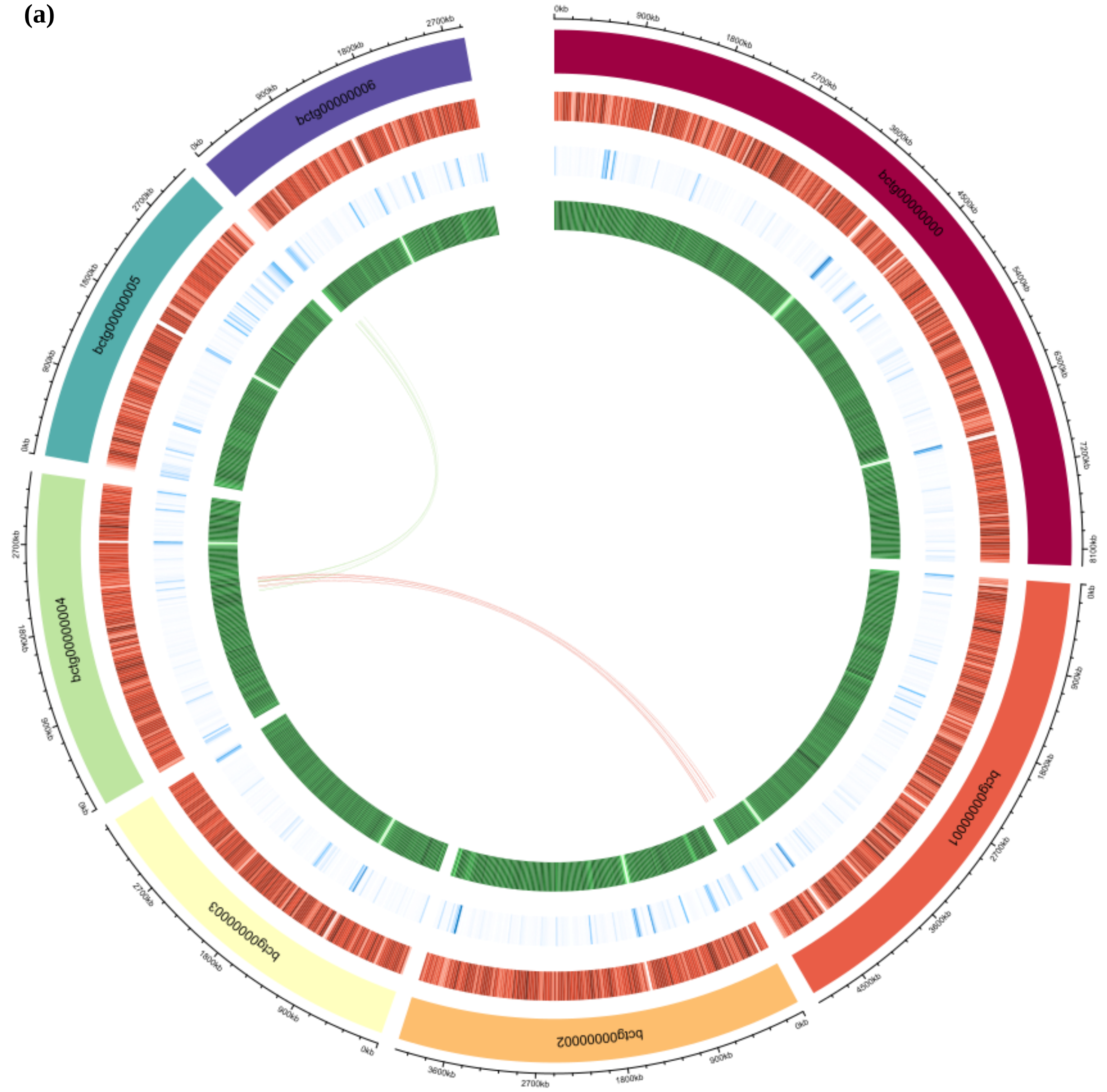
bctg00000000
bctg00000001
bctg00000002
bctg00000003
bctg00000004
bctg00000005
bctg00000006

(b)

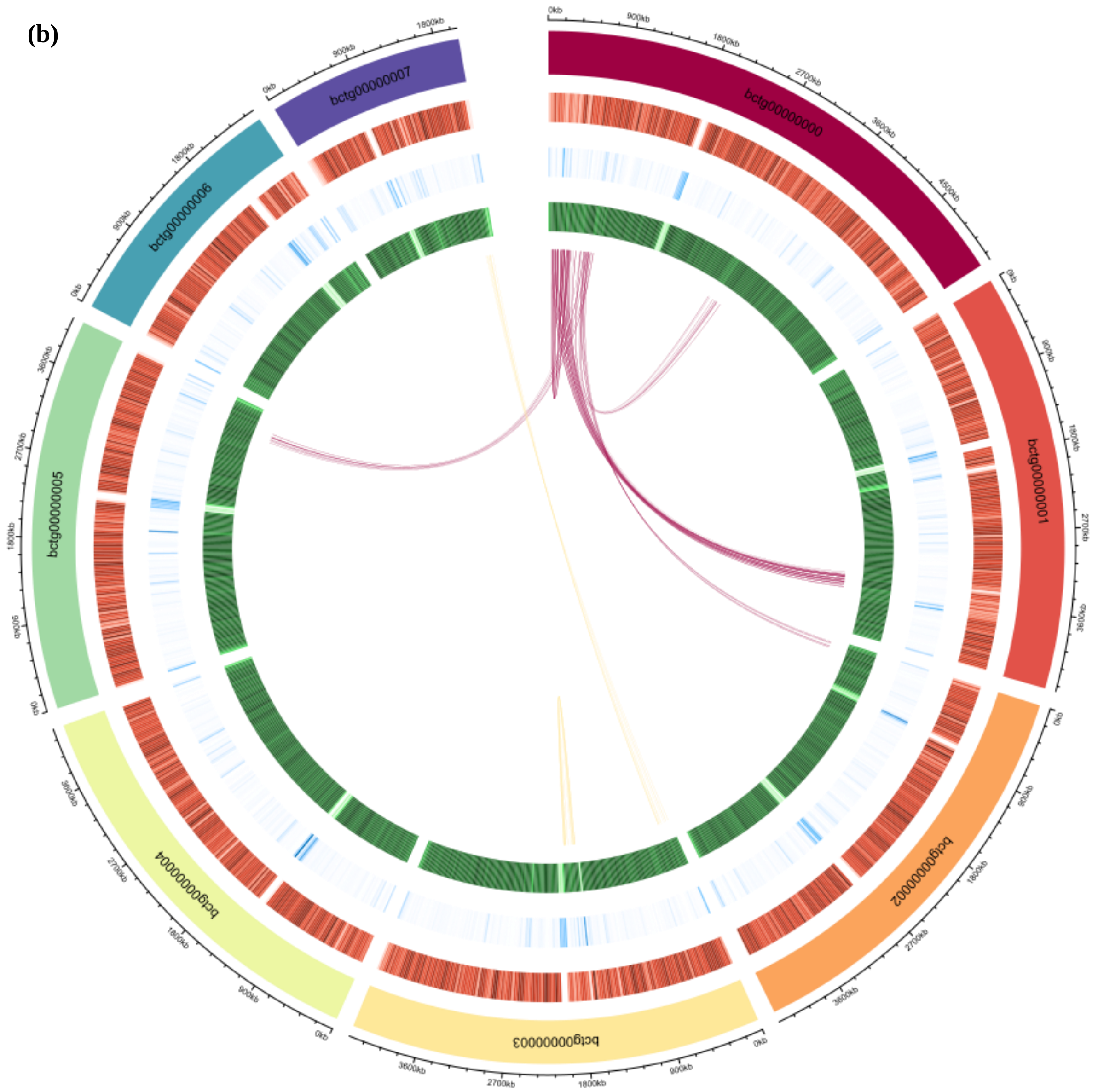
bctg00000000
bctg00000001
bctg00000002
bctg00000003
bctg00000004
bctg00000005
bctg00000006
bctg00000007

(c)

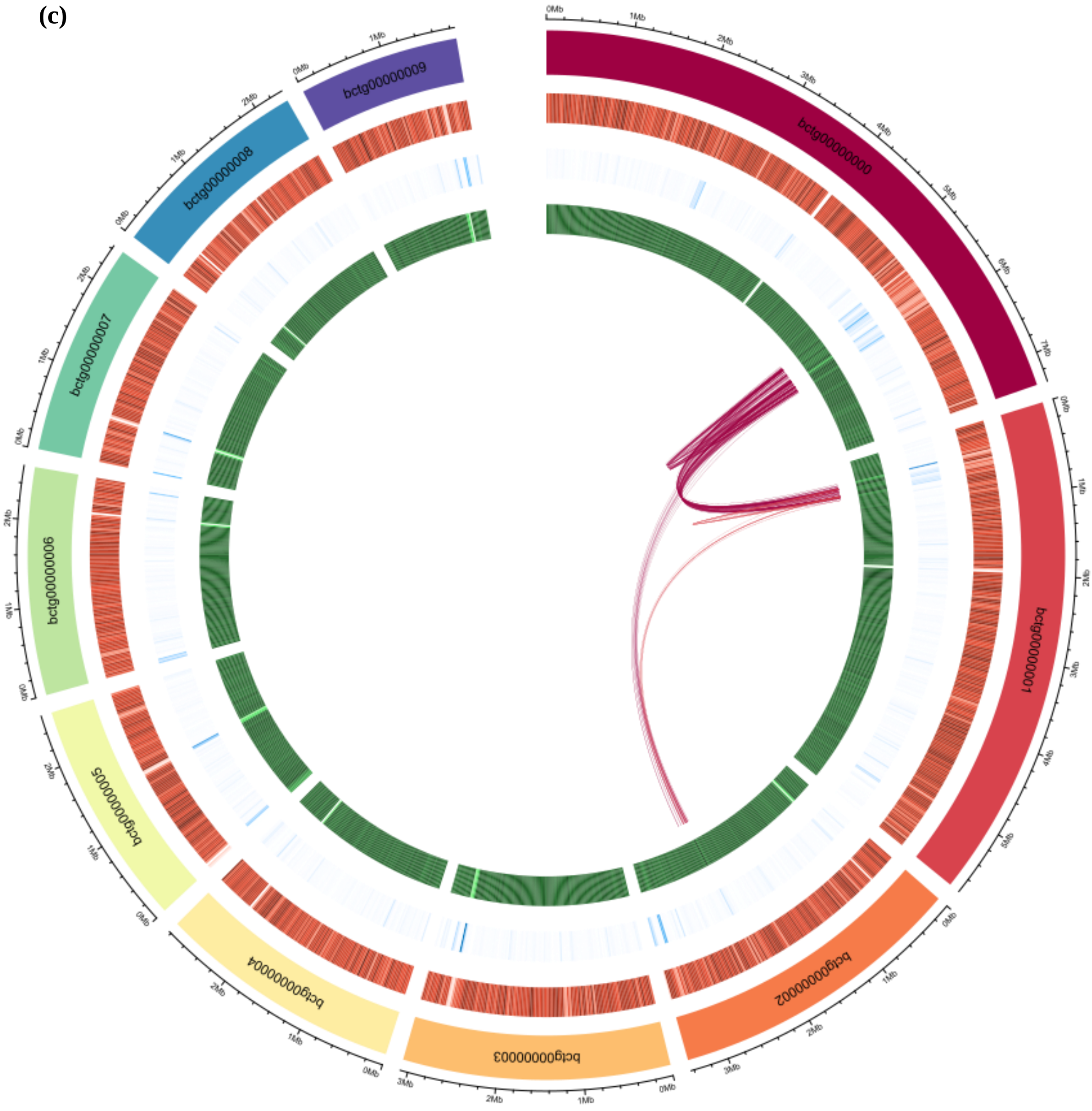

Figure S2. Circular visualization of the complete genomes of three efficient heavy oil-degrading fungi isolated from oil sludge, *Aspergillus corrugatus* FH2 (a), *Aspergillus terreus* FL4 (b), and *Alternaria alstroemeriae* FW1 (c). For the circular chromosomes, circles from the outside to the center indicate: chromosome coordinates, chromosome names, gene density (calculated in 50 kbp windows), repeat sequence density, GC content, and synteny blocks.
